# Fast Differentiation of HepaRG Cells Allowing Hepatitis B and Delta Virus Infections

**DOI:** 10.3390/cells9102288

**Published:** 2020-10-14

**Authors:** Julie Lucifora, Maud Michelet, Anna Salvetti, David Durantel

**Affiliations:** Centre de Recherche en Cancérologie de Lyon (CRCL), UMR Inserm 1052-CNRS 5286, 151 cours Albert Thomas, 69424 Lyon Cedex 03, France; maud.michelet@inserm.fr (M.M.); anna.salvetti@inserm.fr (A.S.); david.durantel@inserm.fr (D.D.)

**Keywords:** Hepatitis D virus, Hepatitis B virus, HepaRG, differentiation

## Abstract

HepaRG cells are liver bipotent progenitors acquiring hepatocytes features when differentiated in the presence of dimethylsulfoxide (DMSO). Differentiated HepaRG (dHepaRG) are considered the best surrogate model to primary human hepatocytes (PHH) and are susceptible to several hepatotropic viruses, including Hepatitis B Virus (HBV) and Hepatitis Delta Virus (HDV) infection. Despite these advantages, HepaRG cells are not widely used for the study of these two viruses because of their long differentiation process and their rather low and variable infection rates. Here, we tested the use of a cocktail of five chemicals (5C) combined or not with DMSO to accelerate the cells’ differentiation process. We found that NTCP-mediated HDV entry and replication are similar in HepaRG cells cultivated for only 1 week with 5C and DMSO or differentiated with the regular 4-week protocol. However, even though the NTCP-mediated HBV entry process seemed similar, cccDNA and subsequent HBV replication markers were lower in HepaRG cells cultivated for 1 week with 5C and DMSO compared to the regular differentiation protocol. In conclusion, we set up a new procedure allowing fast differentiation and efficient HDV-infection of HepaRG cells and identified differential culture conditions that may allow to decipher the mechanism behind the establishment of the HBV minichromosome.

## 1. Introduction

Hepatitis B Virus (HBV) is the second cause of death due to infectious agents worldwide. It is an enveloped virus that infects hepatocytes, the liver parenchymal cells. Hepatitis Delta Virus (HDV) is a satellite virus that uses HBV envelope proteins to egress from infected cells and re-enter into naive cells. HBV particles contain a partially double stranded relaxed DNA (rcDNA) and the virus persists in the nucleus of hepatocytes as a non-integrated episomal double-stranded DNA molecule, named cccDNA. The latter serves as template for the transcription of all HBV RNAs that in turn are translated into viral proteins, including the viral antigens HBeAg and HBsAg. The HDV genome consists of a circular single-stranded negative RNA genome and can produce two HDV antigen (HDAg) isoforms: L-HDAg and S-HDAg [1]. In humans, co-infection with both viruses leads to the most aggressive form of viral hepatitis. It is estimated that among the 250 million people are infected by HBV (WHO data), and approximately 10% are co-infected by HDV. These patients do progress faster to end-stage liver disease compared to HBV-mono-infected ones, with a greater incidence of hepatocellular carcinoma (HCC) [2]. There is still a high need for studies in cell culture models, since current therapies are not curative [3] and the understanding of the interplay between both viruses (i.e., virus/virus/host interactions), which is thought to be responsible for particular pathology, is still elusive.

HBV entry and replication only occur in well/highly differentiated hepatocytes [4]. In contrast, if entry is bypassed, HDV replication is not restricted to hepatocytes [5]. Primary human hepatocytes (PHH) are considered as the gold standard for in vitro HBV/HDV studies [6]. As PHH are not easily accessible, HepaRG cells, which are liver bipotent progenitors [7,8], can be used as an alternative model. In contrast to hepatoma cells (i.e., HepG2 and Huh7 cell lines), which can be used as a model when they overexpress NTCP (Na+ taurocholate co-transporting peptide, the HBV/HDV receptor [9]), HepaRG cells are not transformed and DMSO-induced differentiation promotes the formation of hepatocyte islands within a monolayer of cholangiocytes, with the appearance of functional bile canaliculi and expression of main xenobiotic detoxification/metabolization machinery components [10]. In the absence of HGF (hepatocyte growth factor) and EGF (epithelial growth factor), DMSO-induced differentiation of HepaRG cells leads to 50% of hepatocyte-like cells and 50% of cholangiocyte-like cells [7,11]. Once differentiated, HepaRG display a similar innate immune sensor expression pattern than PHH [12,13], and can be considered as immune competent. Last, but not least, differentiated HepaRG cells (dHepaRG) can be either infected or co-infected by both HBV and HDV [7,14]. In particular, this model was instrumental to depict different interplays between HBV, HDV and innate immune responses, with HDV being described as a strong, MDA5-mediated inducer of the IFN response [15], and HBV as rather silent in this respect [16]. Despite these obvious advantages over hepatoma cell lines, dHepaRG are not widely used mostly because of their long/tedious differentiation process (using 1.8% DMSO), which requires 4 weeks, and their low (approximately 5% to 10% of hepatocyte-like cells) and variable rates of infection by HBV or HDV [7,11].

Different chemicals have been tested recently for their ability to differentiate or maintain differentiated hepatocytes in culture. For instance, a cocktail of five supplements (5C-medium) including Forskolin (an adenylate cyclase inhibitor) combined to SB431542 (a TGF-**β** inhibitor), IWP2 (a Wnt inhibitor), DAPT (a Notch inhibitor) and LDN193189 (an inhibitor of bone morphogenetic protein, BMP) was suggested to help maintain PHH in culture [17]. Forskolin alone was also reported to induce a fast functional polarization of HepaRG [18] and SB431542/DAPT were used to differentiate liver progenitor-like cells in 3D culture conditions and infect them with HBV [19]. Even though we did not find any improvement in the levels of HBV infection in PHH cultivated with 5C-medium [6], we investigated here the use of such chemicals (combined or not with DMSO) to improve the infection of dHepaRG cells with HBV and HDV or accelerate their differentiation process. 

## 2. Materials and Methods

### 2.1. Cell Culture and HBV Infection

Regular differentiation of HepaRG cells and infection with HBV and HDV performed as previously described [7,14]. For fast differentiation, HepaRG cells were seeded and three days later, when confluency was just reached, treated with either FSK (Forskolin, 50 μM, Selleckchem distributed by Euromedex, Souffelweyersheim, France) or 5C cocktail of Forskolin (20 μM, Selleckchem distributed by Euromedex, Souffelweyersheim, France), SB431542 (10 μM, Selleckchem distributed by Euromedex, Souffelweyersheim, France), IWP2 (0.5 μM, Selleckchem distributed by Euromedex, Souffelweyersheim, France), DAPT (5 μM, Selleckchem distributed by Euromedex, Souffelweyersheim, France) and LDN193189 (0.1 μM, Selleckchem distributed by Euromedex, Souffelweyersheim, France) complemented or not with 1.8% DMSO (Sigma-Aldrich, St Quentin, France). HBV inocula were prepared from HepAD38 supernatants [20]. HDV inocula were prepared from supernatants of co-transfected HuH7 cells as previously described [14]. Supernatants containing HBV or HDV particles were concentrated with 8% PEG 8000 (Sigma-Aldrich, St Quentin, France). Viral preparations were tested for the absence of endotoxin (Lonza, Basel, Switzerland).

### 2.2. Nucleic Acid Extractions, Reverse Transcription and qPCR Analyses 

Total intracellular RNAs were extracted from cells with the NucleoSpin RNA II kit (Macherey-Nagel, Hoerdt, France) according to the manufacturer’s instructions. RNA reverse transcription was performed using the Maxima RT (Thermo Scientific™, Life Technologies, Villebon-sur-Yvette, France). Quantitative PCRs were performed using specific primers and normalized to the *PRNP* housekeeping gene (coding for the prion protein) as previously described [14,21]. Protein-free DNAs were extracted with the MasterPure™ Complete DNA and RNA Purification Kit (Epicentre, Lucigene distributed by Euromedex, Souffelweyersheim, France) according to the manufacturer’s instruction except for the proteinase K digestion that was omitted. CccDNA was quantified as previously described using the B-globin as housekeeping gene [22].

### 2.3. Detection of Secreted HBV Antigens 

HBeAg and HBsAg were detected in the supernatant of HBV-infected cells using the Autobio CLIA kit according to the manufacturer (AutoBio, Zhengzhou, China).

### 2.4. Analyses of Intracellular Proteins

For analyses of intracellular proteins, cells were harvested in RIPA lysis buffer (Tris-HCl pH 7.5 10mM, NaCl 140mM, EDTA 1mM, EGTA 0.5mM, 1% Triton X100, 0.1% SDS, 0.1% Na-Deoxycholate) containing protease inhibitors (Complete EDTA-free protease inhibitors from Sigma-Aldrich, St Quentin, France). Clarified lysates were subjected to SDS-PAGE and Western Blot transfer onto nitrocellulose membranes using the iBlot2 apparatus according to the manufacturer’s instructions (Thermo Scientific™, Life Technologies, Villebon-sur-Yvette, France). Anti-HDAg antibodies were produced in house. Detection was performed with Gel Doc XR+ System (BioRad, Marnes-la-Coquette, France) and images were analyzed with ImageJ software. For immunofluorescence (IF) analyses, cells were fixed with 4% paraformaldehyde, permeabilized with 0.1% Triton and then incubated with and anti-albumin antibody (DAKO, A0001) and a secondary Alexa-Fluor 555 antibody (Molecular Probes™, Life Technologies, Villebon-sur-Yvette, France).

### 2.5. Fluorescein Uptake

Medium was replaced by warm medium (-FCS) containing Na-Fluorescein (20 ug/mL, Sigma, St Quentin, France) and cells were incubated for 30 min at 37 °C. Cells were briefly washed with warm PBS and cultured for 5 min at 37 °C in warm medium (-FCS) and then washed two times with PBS before microscopy analyses.

## 3. Results and Discussion

**Figure 1:**
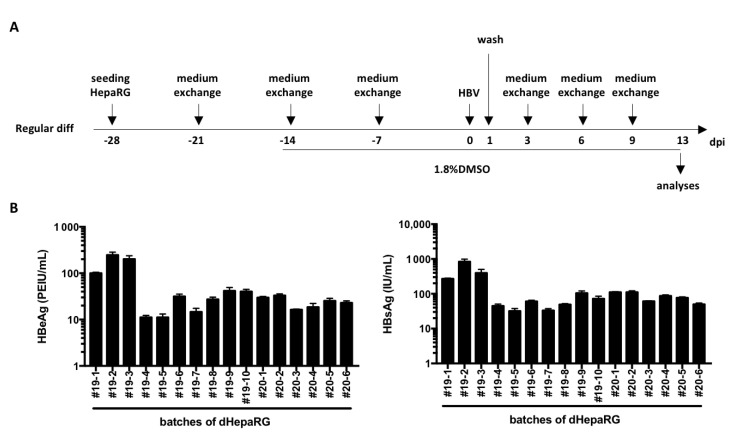
Levels of HBV antigen secreted by HBV-infected dHepaRG. (**A**) HepaRG cells were seeded and differentiated with the regular procedure as indicated. (**B**) At day 13 post-infection (dpi), supernatants were collected from 16 different batches of differentiation and levels of HBeAg and HBsAg were analyzed by ELISA. Bars are the means +/-SD of three biological replicates for each batch.

As mentioned above, HepaRG cells are less used than HepG2-NTCP or HuH7-NTCP cells because of their long differentiation process that requires 4 weeks (Figure 1A) and their low and variable rates of HBV infection [7,11]. Indeed, even though the variation from batch to batch of dHepaRG cells (Figure 1B) is similar to that reported with PHH [6], the mean levels of secreted HBV antigens (55 PEIU/mL for HBeAg and 150 IU/mL for HBsAg) by HBV-infected dHepaRG cells (multiplicity of infection of 500 viral genome equivalent, vge/cells) are, respectively, 34 and 16 times lower on average than those observed for PHH [6].

**Figure 2:**
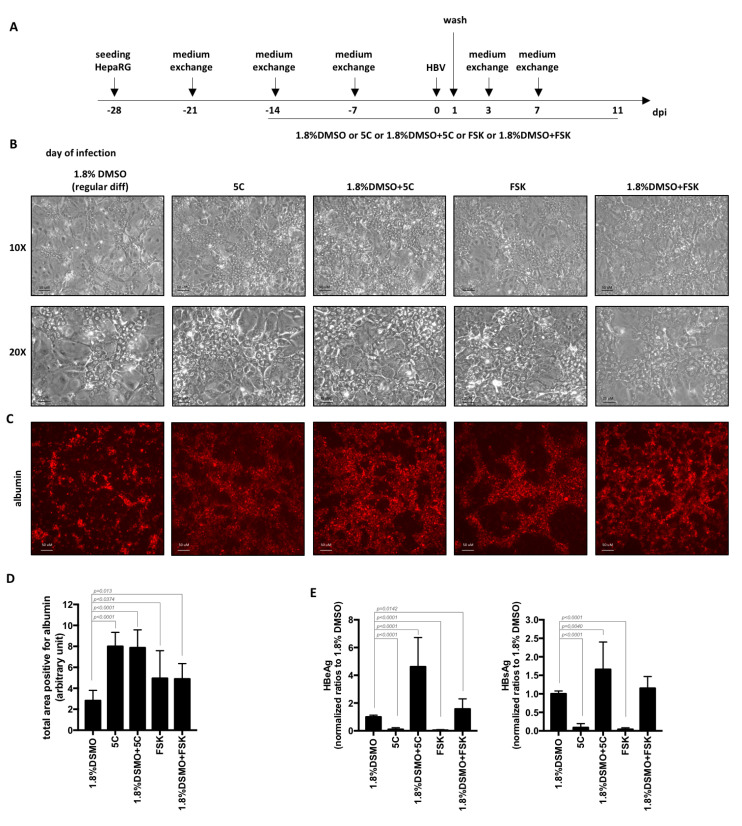
Differentiation of HepaRG with 5C or FSK complemented to 1.8% DMSO increase the levels of secreted HBV antigens. (**A**) HepaRG cells were seeded, treated and HBV-infected (500 vge/cells) as indicated. (**B**–**D**) At the end of the 4 weeks’ differentiation, cells were (**B**) observed by phase contrast microscopy or (**C**,**D**) stained with anti-albumin antibodies. (**D**) Total positive area for albumin were measured using ImageJ. Bars represent the mean +/− SD of 12 fields from two different batches of HepaRG cells. (**E**) Supernatants were collected 7 days post-infections and levels of HBeAg and HBsAg analyzed by ELISA. Bars are the means +/-SD of three independent experiments each performed with three biological replicates. *p* values were calculated using the Prism software and Mann–Whitney analyses.

In order to increase the levels of HBV replication markers, we first tested and compared the use of chemicals contained in the 5C-medium (combined or not with DMSO) to the regular 4-week procedure for the differentiation of HepaRG cells (Figure 2A). Four weeks after seeding, hepatocyte islands appeared larger in cells differentiated in the presence of 5C or FSK (with or without DMSO) compared to cells differentiated with the regular procedure using 1.8% DMSO (Figure 2B). This was confirmed by the quantification of the number of cells expressing albumin, a specific hepatocyte marker, following detection by immunofluorescence (Figure 2C,D). Despite this difference, the use of 5C or FSK in the absence of DMSO for HepaRG differentiation resulted in lower secretions of HBeAg and HBsAg (Figure 2E) compared to the standard differentiation procedure. These results are similar to those we recently reported using primary human hepatocytes cultivated with 5C medium compared to medium containing 1.8% DMSO [6]. However, when combined to DMSO, levels of secreted HBeAg and HBsAg increased by 4.6 and 1.6 times, respectively, compared to regular differentiation with DMSO only (Figure 2E).

**Figure 3:**
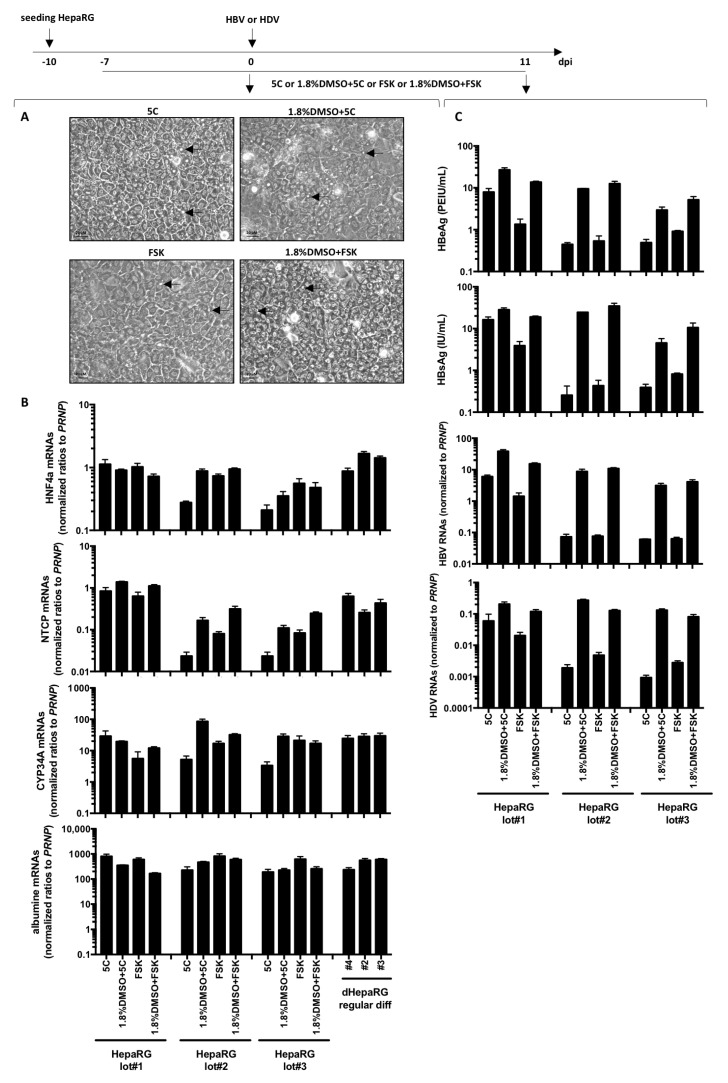
Fast differentiation of HepaRG with chemicals. HepaRG were seeded at low density and treated with 5C, 5C complemented to 1.8% DMSO, FSK or FSK complemented to 1.8% DMSO when confluency was just reached. (**A**) Seven days later, cells were observed by phase contrast microscopy allowing to distinguish refractive bright/white bile canaliculi indicated with arrows on photographies. (**B**) Cells were harvested, and total RNA were extracted. Expression of the indicated hepatocytes differentiation markers was analyzed by RT-qPCR and levels compared to HepaRG differentiated with the regular protocol. (**C**) Cells were infected by HBV or HDV with a multiplicity of infection of 500 vge/cells or 50 vge/cells, respectively. At day 11 post-infection, supernatants were collected and levels of HBeAg and HBsAg analyzed by ELISA. Cells were harvested, RNA extracted and levels of HBV or HDV RNAs were analyzed by RT-qPCR. Results from three independent experiments with three different batches of HepaRG cells are shown and bars are the means +/− SD of three biological replicates per batch.

Next, we investigated the possibility of using 5C and FSK to accelerate and reduce the cost of the HepaRG differentiation process. Interestingly, bile canaliculi (a surrogate marker of differentiation) were already observed in HepaRG treated with 5C, 5C complemented to 1.8% DMSO, FSK or FSK complemented to 1.8% DMSO for 1 week (Figure 3A) and despite variations among cell batches, NTCP, the hepatocyte specific transcription factor HFN4a, the phase-I drug metabolizing enzyme CYP34A and albumin mRNAs were also detected (Figure 3B). 

**Figure 4:**
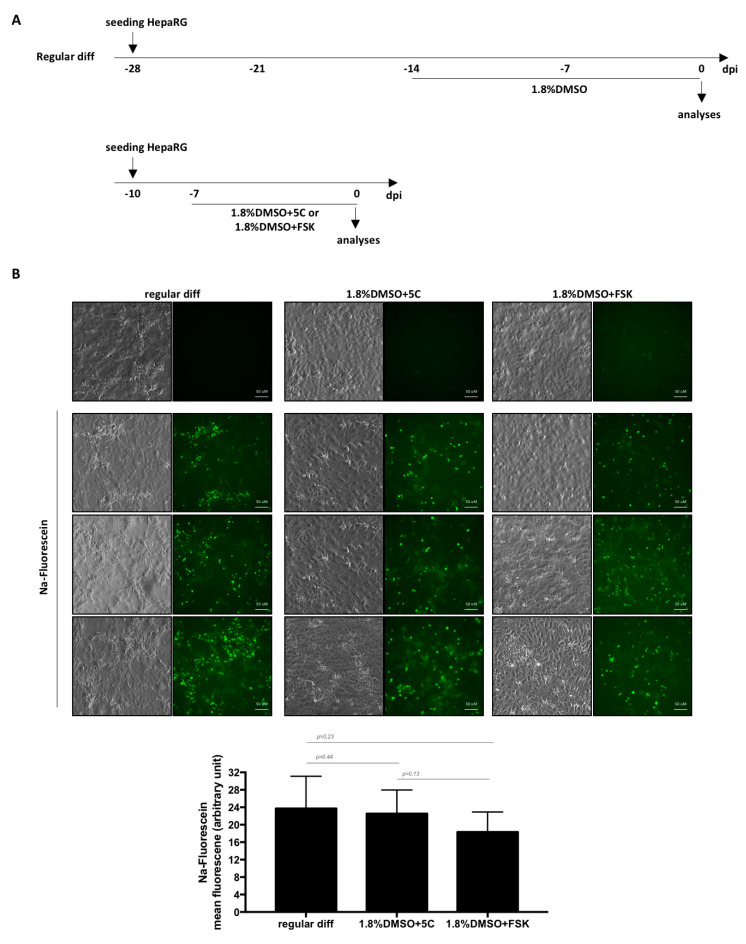
Formation of functional bile canaliculi-like structures in HepaRG. (**A**) Cells were seeded and differentiated as indicated before addition of Na-Fluorescein. (**B**) After a 30 min pulse, cells were briefly washed, chased for 5 min and observed under a fluorescence microscope. Three fields are shown for each condition and the mean fluorescence intensity was measured using ImageJ (lower panel). Bars represent the mean fluorescence +/− SD of 6 to 7 fields. *p* values were calculated using the Prism software and Mann–Whitney analyses.

As NTCP is a key player in HBV/HDV entry [9] and HBV only replicates in highly differentiated hepatocytes, cells were infected with each virus. Productive infections were observed with three batches of HepaRG cells as shown by detectable levels of HBeAg, HBsAg, intracellular HBV or HDV RNAs with all conditions (Figure 3C). However, since fast differentiation of HepaRG cells with 5C or FSK alone leads to very low levels of viral replication markers compared to 5C or FSK combined to DMSO (Figure 3C), we dropped the conditions without DMSO for further investigations. OATP1B1 and OATP1B3 are transporters that are expressed on the basolateral membrane of hepatocytes [23]. The addition of sodium fluorescein, a substrate of OATP1B1 and OATP1B3, to cells treated for 1 week with 1.8%DMSO/5C and 1.8%DMSO/FSK resulted in similar uptake of the dye and a subsequent accumulation in bile canaliculi-like structures as the one observed with a regular 4-week differentiation process (Figure 4A,B) showing that a functional differentiation of the cells was achieved using the fast differentiation procedure.

**Figure 5:**
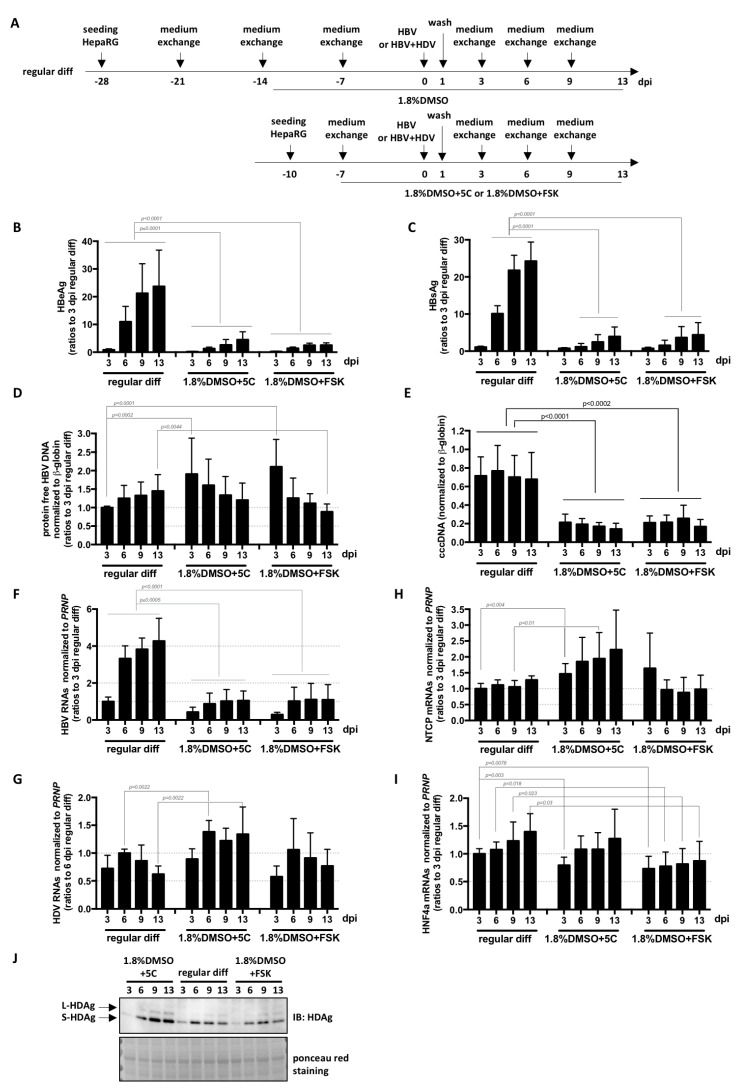
Fast differentiation of HepaRG with 5C or FSK complemented to **1.8**% DMSO allows efficient HDV infection. (**A**) HepaRG cells were seeded and differentiated as indicated, before infection with HBV alone or with HBV and HDV at a multiplicity of infection of 500 vge/cells or 50 vge/cells, respectively. At the indicated day-time post-infection (dpi), supernatants were collected and levels of (**B**) HBeAg and (**C**) HBsAg analyzed by ELISA. Cells were harvested at the same dpi for nucleic acid (RNA, total DNA, and DNA for cccDNA analysis) and protein extractions. Protein-free DNA were extracted and levels of (**D**) total protein-free HBV DNA, as well as (**E**) cccDNA were analyzed by specific qPCR. (**F**) HBV or (**G**) HDV RNA levels, as well as (**H**) NTCP and (**I**) HNF4alpha mRNA level were quantified by RT-qPCR. (**J**) HDV antigens were also analyzed by western blot. Bars are the means +/− SD of three independent experiments each performed with three biological replicates. *p* values were calculated using the Prism software and Mann–Whitney analyses.

Finally, a direct comparison of the kinetics and levels of infection with HBV and HDV was performed on cells submitted to the three differentiation processes (Figure 5A). RNA levels of NTCP and HNF4a mRNAs (both essential for HBV entry and transcription from cccDNA [9,24]) in cells differentiated for 1 week with 1.8%DMSO/5C or with 1.8%DMSO/FSK reached those of cells differentiated for 4 weeks with the regular procedure (Figure 5H,I). Even if the kinetics were similar, the levels of intracellular HDV RNAs and HDAg were slightly higher in HepaRG cells cultivated for 1 week with 1.8%DMSO/5C before infection compared to cells differentiated with the regular protocol (Figure 5G,J). Surprisingly, even though similar levels of intracellular protein-free HBV DNA, mostly corresponding to incoming DNA from virions, are equivalent or even higher (Figure 5D),were measured among the three conditions, cccDNA levels (Figure 5E) and all subsequent HBV replication markers (intracellular HBV RNAs, and secreted HBeAg and HBsAg, Figure 5B,C,H) were lower in cells cultivated for 1 week with 1.8%DMSO/5C or with 1.8%DMSO/FSK compared to those differentiated following the regular protocol. Altogether, these data suggest that (1) NTCP-mediated HDV and HBV entry process is similar in HepaRG cells cultivated for 1 week with 1.8%DMSO/5C, with 1.8%DMSO/FSK or differentiated with the regular protocol, (2) one or several host factor(s) required for/or preventing conversion of rcDNA into cccDNA is(are) expressed at lower/higher levels in HepaRG cells cultivated for 1 week with 1.8%DMSO/5C or with 1.8%DMSO/FSK. Whether the slight increase in the levels of intracellular HDV RNAs and proteins observed in cells cultivated with 1.8%DMSO/5C (Figure 5G,J) is the result of low HBsAg production (Figure 5C) and a subsequent defect of HDV particle secretion remains to be determined. Additionally, the precise mode of action of 5C (or FSK alone) and DMSO should also be investigated. Our data suggest that DMSO on one hand, and the combination of the other five chemicals, on the other hand, probably influence cells differentiation at different/complementary stages since combining them all seems beneficial. It would be interesting to test if host factors important for HBV, such as the newly identified one CDKN2C [25], are differentially influenced by DMSO and 5C, and if additional ones could be uncovered.

## 4. Conclusions

We set up a new procedure allowing fast differentiation and efficient HDV-infection of HepaRG cells. Moreover, we identified differential culture conditions that may be used to investigate the mechanism behind rcDNA to cccDNA conversion. Indeed, this step relies on the cellular DNA repair machinery, and cell culture conditions may largely influence this process. Cell-based genetic approaches, using siRNA or Crispr/Cas9, are difficult to implement because of the importance of the DNA repair proteins in cell survival and in vitro biochemical systems have been used to mimic rcDNA to cccDNA [26,27]. We propose to use these different culture conditions as a complementary approach to confirm the involvement of cellular factors in this process [26] and possibly discover additional ones.
